# Esketamine in patient with treatment resistant depression: Outcome of the temporary authorization for use programme in France

**DOI:** 10.1192/j.eurpsy.2021.1834

**Published:** 2021-08-13

**Authors:** E. Gaudre Wattinne, L. Mekaoui, M. Rothärmel, M.A. Codet, S. Bouju

**Affiliations:** 1 Neurosciences, JANSSEN CILAG, ISSY LES MOULINEAUX, France; 2 Service Du Pr P. Gorwood, Clinique Des Maladies Mentales Et De L’encéphale (cmme), Centre hospitalier Sainte-Anne, Paris cedex, France; 3 University Department Of Psychiatry, Centre Hospitalier du Rouvray, Sotteville-lès-Rouen, France

**Keywords:** treatment resistant depression, spray nasal, glutamatergic pathway, esketamine

## Abstract

**Introduction:**

Esketamine nasal spray has been developed for patients with treatment resistant depression.

**Objectives:**

A cohort Temporary Authorization for Use (ATUc) allowed to collect for a 6-month period the first data in real life

**Methods:**

On 02/08/2019 the French National Agency for Medicines and Health Product Safety granted an early access program for Esketamine nasal spray framed by a specific protocol for patients without therapeutic alternatives. Each treatment request was approved based on inclusion and exclusion criteria. Clinical evolution, treatment management and safety were then spontaneously reported by psychiatrists.

**Results:**

From 09/23/2019 to 03/25/2020, 66 patients were treated. The median age was 53 years and 41 (62.1%) were females. At treatment request, 52 patients (79%), presented a severe current depressive episode based on clinical judgment. The median duration of the disease was 12.2 years and the current episode was 2.6 years. Since the beginning of the current depressive episode, all patients (66) were prescribed ≥2 antidepressants (mean 4.2). Esketamine was initiated in a complete hospitalization setting in 27 patients (55.1%) and in day hospitalization in 22 patients (44.9%).Safety profile was consistent with the one described during clinical study. The most frequently adverse events reported (>10%) were dizziness, sedation, sleepiness, anxiety and dissociation. Most of them appeared after treatment administration and were transient.

**Conclusions:**

ATUc ended on 12/18/2019 after Marketing Authorization granted by European Medicines Agency. Data reported by French psychiatrists are the first collected in this specific population and provide descriptive information on patient characteristics, burden of disease; Esketamine management and practical use at hospital level

**Disclosure:**

Data analysis performed by RCTs and poster conception coordinated by Medergy and funded by Janssen

